# Optimal Preparation and Performance Study of Eco-Friendly Composite Chemical Dust Suppressants: A Case Study in a Construction Site in Chengdu

**DOI:** 10.3390/ma17102346

**Published:** 2024-05-15

**Authors:** Yong Xu, Ben Ma, Yingda Zhang, Yujie Fan

**Affiliations:** School of Architecture and Civil Engineering, Xihua University, Chengdu 610039, China

**Keywords:** dust pollution, response surface methodology, green environmental protection, bonding performance

## Abstract

To mitigate dust pollution generated during various stages of construction activities and reduce the environmental and health hazards posed by airborne dust, this study utilized hydroxyethyl cellulose, glycerol, and isomeric tridecyl alcohol polyoxyethylene ether as raw materials to formulate a composite chemical dust suppressant. The properties of the dust suppressant were characterized through analysis. Employing single-factor experiments, the optimal proportions of the binder, water-retaining agent, and surfactant for the composite dust suppressant were determined. Subsequently, a response surface model was established, and, after analysis and optimization, the optimal mass ratios of each component in the composite dust suppressant were obtained. Under optimal ratios, the physicochemical properties and wind erosion resistance of the composite dust suppressant were analyzed. Finally, the practical application of the suppressant was validated through on-site trials at a construction site. This study revealed that the optimal formulation for the dust suppressant was as follows: 0.2% hydroxyethyl cellulose, 2.097% glycerol, 0.693% isomeric tridecyl alcohol polyoxyethylene ether, and the remainder was pure water. The suppressant is non-toxic, non-corrosive, environmentally friendly, and exhibits excellent moisture retention and bonding properties compared to water. The research findings provide valuable insights for addressing dust pollution issues on construction sites.

## 1. Introduction

Construction operations on building sites are notorious sources of atmospheric pollution, generating varying degrees of dust pollution at different stages of construction, which adversely affects both human health and the ambient air quality [1]. Dust refers to a general term for solid particles that can persist in the air for an extended period [2]. Airborne dust often contains numerous toxic components, such as chromium, manganese, cadmium, lead, mercury, arsenic, and more [3]. When individuals inhale dust, particularly particles smaller than 5 μm, these particles can easily penetrate deep into the lungs, causing toxic pneumonitis or silicosis, and, in some cases, even leading to lung cancer [4]. Pollutants deposited in the lungs, once dissolved, directly enter the bloodstream, causing blood poisoning. Undissolved pollutants may also be absorbed by cells, leading to structural damage to the cells.

Existing research indicates that earthwork, specifically excavation and filling, is the most significant contributor to pollutant emissions throughout the entire construction process [5]. Dust, characterized by its wide coverage and rapid dispersion, poses challenges in pollution control, as pollutants can remain suspended in the air for extended periods without settling [6]. The issue of dust pollution control during construction operations urgently requires attention. Currently, mainstream dust control methods include electric dustproof technology, mist dust removal technology, and environmentally friendly dust suppressant technology, among others [7].

Wang et al. [8], by incorporating water-retaining agents and surfactants into binders, discovered a significant improvement in the hardness and water retention of the dust suppressant composite. Hu et al. [9] formulated a frost-resistant dust suppressant, enhancing water retention performance by 88%. Yu et al. [10] used humic acid (HA) and grafted acrylamide (AM) as the main raw material, grafting it to produce a dust suppressant for coal transportation, which showed non-corrosive properties.

With increasing environmental demands, dust suppressants are evolving towards functional composites, emphasizing green, environmentally friendly, and degradable characteristics [11]. Eco-friendly composite chemical dust suppressants are composed of binders, water-absorbing agents, and water-retaining agents, each derived from environmentally friendly and degradable materials, thus avoiding secondary pollution [12]. Feng et al. [13] utilized peanut shells to prepare a highly effective and environmentally friendly novel degradable nanocellulose dust suppressant. Compared to traditional dust suppressants, eco-friendly composite dust suppressants offer more comprehensive functionality and longer-lasting dust suppression, with the added benefit of cost reduction. Tripathi and Sandha [14] used polyvinyl alcohol as a monomer, ammonium persulfate as an initiator, aluminum hydroxide as a cross-linking agent, and glycerol as a plasticizer to prepare a polyvinyl alcohol-grafted cellulose-based sugarcane bagasse dust suppressant in a microwave reactor, significantly reducing production costs.

Compound dust suppressants have substantially reduced costs and can fundamentally address dust pollution issues on construction sites. For instance, Lee et al. [15] utilized environmentally friendly methylcellulose-based polymers to investigate particulate matter reduction efficiency. Medeiros et al. [16] used glycerol oligomerization to produce dust suppressants and the test results showed that hexa and heptaglycerol exhibited a good viscosity and dust suppression performance. Moreover, Gao et al. [17] adopted the response surface method (RSM) to evaluate the dust reduction effect. The above studies provide scientific evidence to select the proposed materials and method for dust suppressant preparation in this study.

Therefore, this study aligns with the principles of green development and proposes the development of an eco-friendly composite chemical dust suppressant using hydroxyethyl cellulose (HEC), glycerol (C_3_H_8_O_3_), and isomeric tridecyl alcohol polyoxyethylene ether (AEO-13) as raw materials. This dust suppressant is designed to efficiently control dust through film binding, water retention, and high wetting properties, exhibiting multifunctionality. Through response surface methodology, a response surface model is established to analyze the interrelationships and degrees of influence among various components, thereby obtaining the optimal mixing ratio of dust suppressant components. The feasibility of dust suppression is verified through microscopic characterization, compositional analysis, and practical applications at construction sites, providing a new approach to the prevention and control of dust pollution at construction sites.

## 2. Experimental Program

### 2.1. Raw Materials

Materials used in this experiment include hydroxyethyl cellulose, sodium polyacrylate, propylene glycol, triethanolamine, Sodium Dodecyl Sulfate, Sodium Dodecyl Benzene Sulfonate, and Isostearyl Alcohol Ethoxylate, all analytical grade. Reagents were purchased from Shandong Youso Chemical Technology Co., Ltd. (Linyi, China), Tianjin Kemiou Chemical Reagent Co., Ltd. (Tianjin, China), Changde Bickman Biotechnology Co., Ltd. (Changde, China), and Wuxi Jingke Chemical Co., Ltd. (Wuxi, China). RO water was prepared in house.

### 2.2. Test Instruments

The experimental apparatus includes an FA2004B electronic analytical balance from Foshan Nanbeihu E-Commerce Co., Ltd. (Foshan, China), an HN101-3 blast drying oven from Nantong Hunan Scientific Instrument Co., Ltd. (Nantong, China), an HJ-4A multi-head magnetic heating stirrer from Jinan Oulaibo Scientific Instrument Co., Ltd. (Jinan, China), an HHWZI-600 constant temperature and humidity water bath from Henan Shuli Instrument Co., Ltd. (Zhengzhou, China), a PHB-5 digital pH meter from Shanghai Yidian Scientific Instruments Co., Ltd (Shanghai, China), an NDJ-5S viscometer from Shanghai MiTong Mechanical and Electrical Technology Co., Ltd. (Shanghai, China), and an Apreo 2C scanning electron microscope from Shanghai Thermo Fisher Scientific Company (Shanghai, China). Additionally, several items, such as beakers, standard sieve screens, evaporating dishes, glass rods, etc., were also used. 

### 2.3. Test Procedures

The developed dust suppressant in this study is intended for dust control in construction sites. Combining with the mechanism of dust suppressant agents, the specific experimental procedures are outlined as follows:

First are single-factor optimization experiments for the composite dust suppressant [18]. Through single-factor experiments, adhesive agents, water-retaining agents, and surfactants are screened from various functional additives to formulate an environmentally friendly composite dust suppressant. The corresponding dust suppression performance data were used as screening indicators to explore the stability of the performance of each auxiliary agent and determine the optimal concentration range.

Second, orthogonal optimization experiments are based on response surface methodology. The viscosity agent, water-retaining agent, and surfactant selected from the single-factor experiments were used as independent variables. Viscosity, evaporation resistance, and permeability rate were taken as response values. A Box–Behnken model was established for experimental design, optimizing the values of each component of the dust suppressant [19]. Subsequently, through variance analysis, two-factor interaction analysis, model validation, and experimental verification, the results showed good agreement between the predicted values of the model and the experimental values, proving that the calculated optimal ratio of the model is reasonable and effective.

**Figure 1:**
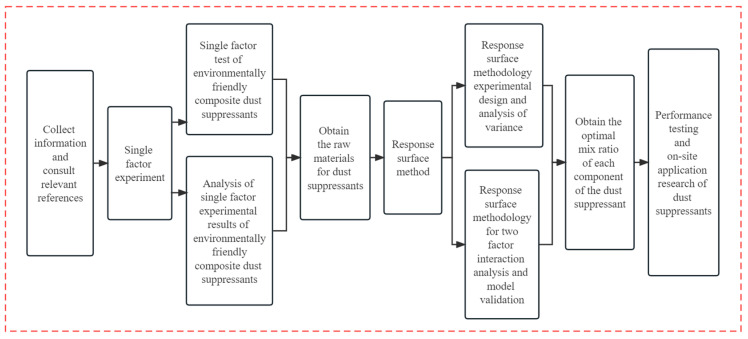
Technology roadmap.

Third, characterization of the dust suppressant properties and wind erosion resistance testing [20]. Property characterization includes pH value, surface tension, viscosity, determination of toxic and harmful substances, and SEM scanning electron microscope microscopic morphology analysis. A comprehensive evaluation was conducted from both the physicochemical indicators including viscosity, water retention rate, surface tension, and morphological characteristics of the dust suppressant. Wind erosion resistance testing aimed to demonstrate the ability of the composite dust suppressant to withstand adverse weather conditions. The experimental method flowchart is presented in Figure 1.

## 3. Selection of Dust Suppressant Raw Materials

### 3.1. Selection and Treatment of Soil Samples

The experimental test conducted a sampling survey at a construction site [20]. The sampling method followed the snake-shaped sampling technique used in soil sampling, with a total of nine sampling points established [21]. A specific area in the construction site was chosen, and sampling points were evenly distributed in a “Z” pattern. During sampling, the surface soil within a 5-square-meter area around each sampling point was swept with a broom and collected in a soil collection cylinder. After sampling all nine points, the collected samples were transported back to the laboratory for further use.

For dust sample processing, larger particles such as sand, gravel, dry branches, and plastic waste were initially removed as construction debris. Subsequently, the dust samples underwent grinding treatment. The ground dust samples were sieved through a 100-mesh standard sieve, and the sieved dust was dried in a constant-temperature forced-air drying oven at 105 °C for 8 h. Afterward, the samples were cooled to room temperature in a drying chamber, weighed, packaged, and stored for future use.

### 3.2. Optimal Selection of Binder

**Figure 2:**
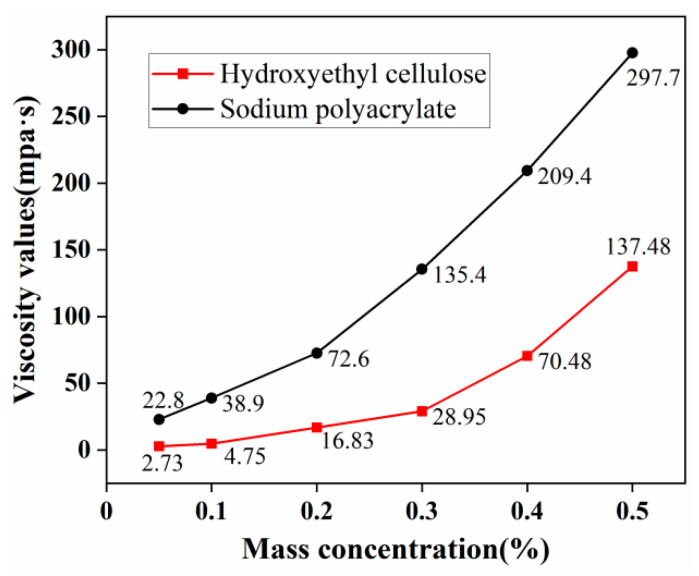
Viscosity values at different concentrations for two binders (25 °C).

The bond strength test is a crucial indicator for evaluating the dust suppression effectiveness of dust suppressants. Some scholars, when optimizing binders, have utilized hydroxyethyl cellulose and sodium polyacrylate separately to formulate dust suppressants. Both hydroxyethyl cellulose [22] and sodium polyacrylate [23] exhibit excellent bonding properties without causing environmental pollution. Therefore, building upon this foundation, this experiment further optimized between the two materials. Hydroxyethyl cellulose and sodium polyacrylate solutions were prepared with concentration gradients of 0.05%, 0.1%, 0.2%, 0.3%, 0.4%, and 0.5% by mass. Viscosity measurements and temperature sensitivity tests were conducted to optimize these materials as binders for the composite dust suppressant. The viscosity measurement results are depicted in Figure 2.

Viscosity measurements were conducted for hydroxyethyl cellulose and sodium polyacrylate, and the results are presented in Figure 1. At a mass concentration of 0.05%, the viscosity of hydroxyethyl cellulose is 2.73 mPa·s, which increases to 137.48 mPa·s as the mass concentration rises to 0.5%. Higher viscosity values promote particle aggregation, but for practical spraying convenience, a moderate viscosity is desirable. Hence, hydroxyethyl cellulose concentrations in the range of 0.2% to 0.4% were selected for response surface optimization analysis.

Additionally, it was observed that at a mass concentration of 0.05%, the viscosity of sodium polyacrylate is 22.8 mPa·s, reaching 297.7 mPa·s as the mass concentration increases to 0.5%. Sodium polyacrylate exhibits good bonding properties at lower concentrations but experiences reduced sprayability at higher concentrations, diminishing its practicality.

**Figure 3:**
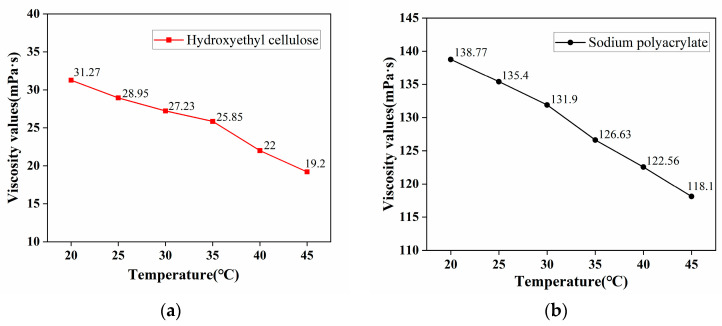
The viscosity values at different temperatures for (**a**) hydroxyethyl cellulose; (**b**) sodium polyacrylate.

Subsequently, this study conducted temperature sensitivity tests on hydroxyethyl cellulose and sodium polyacrylate. Temperature, serving as the sole independent variable, was set at gradients of 20 °C, 25 °C, 30 °C, 35 °C, 40 °C, and 45 °C. Test experiments were carried out using solutions of hydroxyethyl cellulose and sodium polyacrylate with a mass concentration midpoint of 0.3% from the previously mentioned concentration gradients. The experimental results are presented in Figure 3.

From the temperature sensitivity test results, it can be observed that as the temperature increases, molecular movement accelerates, leading to a decrease in viscosity. The viscosity of hydroxyethyl cellulose solution decreases from 31.27 mPa·s to 19.2 mPa·s, while the viscosity of sodium polyacrylate solution decreases from 138.77 mPa·s to 118.1 mPa·s. It is evident that the hydroxyethyl cellulose solution is more stable in high-temperature environments. Since this dust suppressant is primarily intended for outdoor open spaces with varying temperatures during outdoor operations, a concentration range of 0.2% to 0.4% for hydroxyethyl cellulose was ultimately selected as the binder for the environmentally friendly composite dust suppressant for further response surface optimization analysis.

### 3.3. Optimal Selection of Water-Retaining Agent

Water retention performance is also a crucial indicator for assessing dust suppression effectiveness. In this study, we selected two commonly used water-retaining agents, glycerol and triethanolamine. Solutions of triethanolamine and glycerol were prepared with mass concentrations of 0.01%, 0.05%, 0.1%, 0.5%, 1%, and 3%. Additionally, a control experiment using plain water was set up. Using a spraying quantity of 15 mL, the prepared water-retaining agent solutions were sprayed onto aluminum boxes containing soil samples. Subsequently, the aluminum boxes were left to naturally evaporate under room temperature conditions. The experimental results are presented in Table 1 and Table 2.

From the water retention rate results of glycerol and triethanolamine, it can be observed that the water retention rate of the 3% glycerol solution after 144 h is 12.83%, while the water retention rate for the control group with plain water is only 2.37% after 144 h. The water retention rate depends on the water-absorbing capacity of the water-retaining agent, and particles infiltrated with water are less likely to become airborne in the atmosphere. Glycerol exhibits excellent water retention capacity, and when the mass concentration exceeds 3%, the water retention rate of glycerol stabilizes at around 13%. Therefore, the concentration range of 1% to 3% was selected for response surface optimization. Additionally, the water retention rate of the 3% triethanolamine solution after 144 h is 9.1%, which is higher than the control group with plain water but inferior to the glycerol solution in terms of water retention performance. Hence, glycerol solution was chosen as the water-retaining agent for the environmentally friendly composite dust suppressant.

### 3.4. Optimal Selection of Surfactant

**Figure 4:**
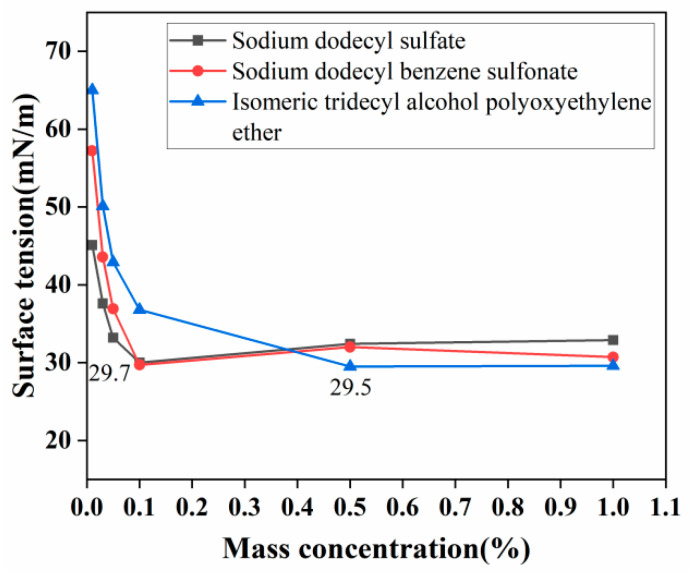
Surface tension curves of three solutions.

The primary reason for choosing surfactants is based on their excellent wetting and emulsifying properties, effectively reducing the surface tension of the dust suppressant solution. When surfactants are dissolved in water, the hydrophilic groups facing the water side reduce the surface tension of the solution, enhancing the ability of the composite dust suppressant to wet the dust. The hydrophobic groups on the opposite side facing away from water form the interfacial adsorption forces that can effectively capture airborne dust particles. Additionally, due to their emulsifying effect, the solution of the composite dust suppressant disperses well and is less prone to precipitation, facilitating spraying [24]. For these reasons, this experiment selected three surfactants with good wetting and emulsifying properties—Isotridecanol Polyethylene Glycol Ether (M1), Sodium Dodecyl Sulfate (M2), and Sodium Dodecyl Benzene Sulfonate (M3)—to conduct surface tension testing. The test results are shown in Figure 4.

From Figure 4, it can be observed that Isotridecanol Polyethylene Glycol Ether (M1) reaches a surface tension of 29.5 mN/m at a mass concentration of 0.5%. A lower surface tension indicates better wetting performance, leading to improved dust suppression performance in practical applications. Additionally, after the mass concentration of Isotridecanol Polyethylene Glycol Ether (M1) reaches 0.5%, the surface tension stabilizes around 29.5 mN/m. Therefore, for the dust suppressant, Isotridecanol Polyethylene Glycol Ether (M1) was chosen as the surfactant, and the concentration range of 0.5% to 1% was selected for response surface optimization.

## 4. Optimal Selection of Dust Suppressant Mix Design

### 4.1. Mix Design Using Response Surface Methodology

After the preliminary single-factor experiments, the environmentally friendly composite dust suppressant was tentatively composed of a binder—hydroxyethyl cellulose (HEC) solution; a water-retaining agent—glycerol (C_3_H_8_O_3_) solution; and a surfactant—Isotridecanol Polyethylene Glycol Ether (AEO-13) solution, with the remainder being pure water. Considering the interaction among the additives and exploring the optimal combination among them, the response surface methodology (RSM) was employed to determine the best ratio of each additive level. Based on the analysis of the single-factor experimental results, a three-factor, three-level experiment was determined. Due to the relatively low number of factor levels in this experiment and the fewer required experimental runs compared to the Central Composite Design (CCD) [25] under the same factors, the Box–Behnken Design (BBD) was chosen for experimental design [26]. The three factors were hydroxyethyl cellulose, glycerol, and Isotridecanol Polyethylene Glycol Ether. The three response variables corresponded to viscosity, evaporation resistance, and permeability rate. The details of the experimental design are shown in Table 3.

### 4.2. Variance of Regression Model Analysis

#### 4.2.1. Viscosity

The variance analysis of the response factor viscosity is shown in Table 4. A, B, and C represent HEC, C_3_H_8_O_3_, and AEO-13, respectively. Note that in statistics, the F-value serves as a measure for variance analysis, where a higher F-value indicates a better fit between the model and the data. The *p*-value, on the other hand, is utilized to assess the significance of statistical test results. A smaller *p*-value suggests that the observed results are less likely to be caused by random factors. From Table 4, it can be observed that the F-value of the viscosity response model is 92.76, with *p* < 0.0001, indicating a highly significant impact of the independent variables on viscosity. The lack-of-fit F-value is 3.35, with *p* = 0.1368 > 0.05, suggesting that the lack of fit is not significant, and the model has a small deviation between predicted values and experimental data. The determination coefficient R^2^ is 0.9917, the adjusted determination coefficient Radj^2^ is 0.9810, the predicted determination coefficient Rpre^2^ is 0.9012, and |Radj^2^ − Rpre^2^| < 0.2. The signal-to-noise ratio AP is 29.7126 > 4, indicating a strong response signal, and the impact of random errors is minimal on the model.

#### 4.2.2. Evaporation Resistance

The variance analysis of the response factor, evaporation resistance, is shown in Table 5. It can be observed that the F-value of the evaporation resistance response model is 86.66, with *p* < 0.0001, indicating a highly significant impact of the independent variables on evaporation resistance. The lack-of-fit F-value is 4.21, with *p* = 0.0995 > 0.05, suggesting that the lack of fit has an insignificant impact, and the model’s predicted values show a small deviation from the experimental data. The model’s determination coefficient R^2^ = 0.9911, the corrected determination coefficient Radj^2^ = 0.9797, the predicted determination coefficient Rpre^2^ = 0.8886, and |Radj^2^ − Rpre^2^| < 0.2. The signal-to-noise ratio AP = 32.9945 > 4, indicating a strong response signal and minimal impact from random errors, demonstrating the overall high fitting degree of the model.

#### 4.2.3. Permeability Rate

The analysis of variance for the response factor, permeability rate, is presented in Table 6. The F-value of 79.14 with *p* < 0.0001 indicates a highly significant impact of the independent variables on the permeability rate. The lack-of-fit F-value is 2.98 with *p* = 0.1597 > 0.05, suggesting that the impact of the lack of fit is not significant, and the model’s predicted values show a small deviation from the experimental data. The model’s determination coefficient (R^2^) is 0.9903, with the adjusted determination coefficient (Radj^2^) at 0.9778 and the predicted determination coefficient (Rpre^2^) at 0.8878, where |Radj^2^ − Rpre^2^| < 0.2. The signal-to-noise ratio (AP) is 34.5443, which is greater than 4, indicating that the model has a strong response signal, and occasional errors have minimal impact on it. This suggests a high overall fit of the model.

#### 4.2.4. Two-Factor Interaction Analysis

To further investigate the interaction effects among hydroxyethyl cellulose (HEC), glycerol, and isooctylphenol polyoxyethylene ether (AEO-13) on the viscosity, anti-evaporation, and permeability of the mixed solution, aiming to obtain the optimal ratio for an environmentally friendly composite dust suppressant, Design Expert software (Version 13.0.5.0) was utilized [27]. Two factors were held constant while plotting 3D response surface and contour plots for the other two factors, providing a visual analysis of the strength and significance of the interaction effects between the factors and their impact on the dust suppressant’s performance.

**Figure 5:**
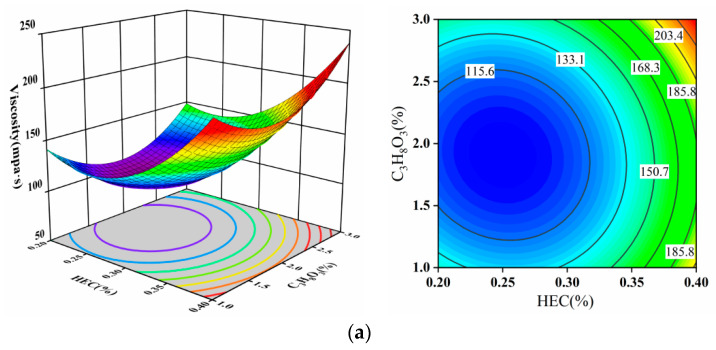

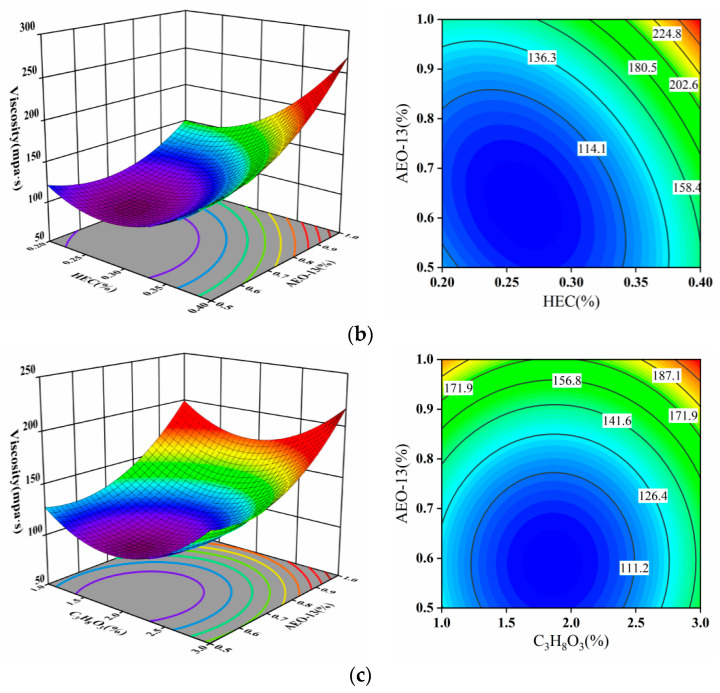
(**a**) HEC and C_3_H_8_O_3_; (**b**) HEC and AEO-13; (**c**) C_3_H_8_O_3_ and AEO-13 effect on viscosity using 3D surface and contour plots.

From the 3D surface plots in Figure 5, it is evident that the response surfaces for viscosity (AB, AC, BC) exhibit significant curvature, with colors shifting from blue to red indicating substantial variations. This implies a substantial impact of the interaction between hydroxyethyl cellulose (HEC) and glycerol (C_3_H_8_O_3_) or isooctylphenol polyoxyethylene ether (AEO-13) on viscosity. The contour plots for AB, AC, and BC all display elliptical shapes, indicating a significant interaction between these factors. In Figure 5a, when the mass concentration of HEC is 0.25%, the viscosity initially decreases and then increases as the concentration of C_3_H_8_O_3_ increases, with the amplitude first being small and then becoming larger. In Figure 5b, when the mass concentration of HEC is 0.3%, the viscosity follows a similar trend as the AEO-13 concentration increases. In Figure 5c, with C_3_H_8_O_3_ concentration at 2%, the viscosity decreases and then increases with an increase in AEO-13 concentration, showing a similar pattern of smaller and then larger amplitudes.

**Figure 6:**
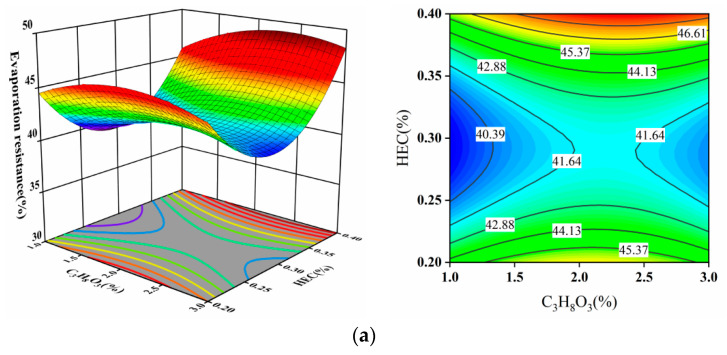

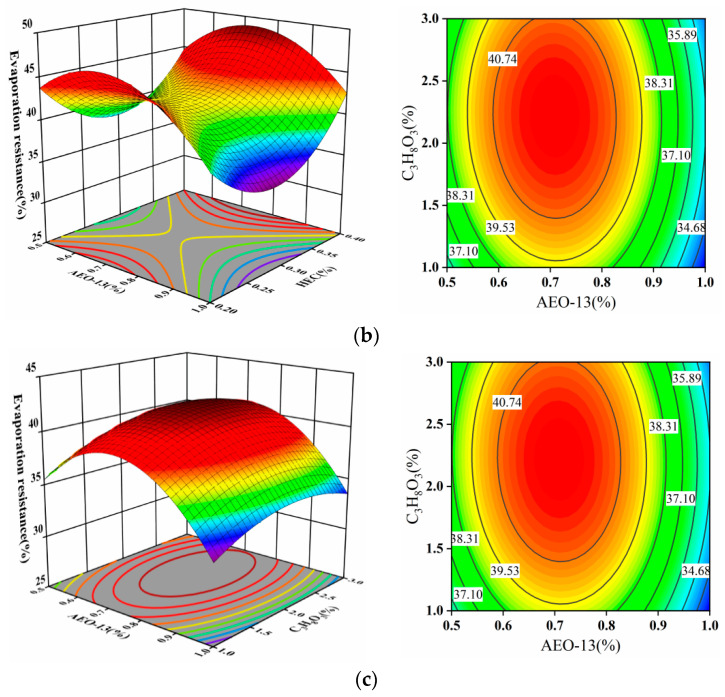
(**a**) HEC and C_3_H_8_O_3_; (**b**) HEC and AEO-13; (**c**) C_3_H_8_O_3_ and AEO-13 effects on evaporation resistance using 3D surface and contour plots.

The 3D surface plots in Figure 6 reveal that the response surface for evaporation resistance (BC) exhibits significant curvature with a steep slope, indicating a highly significant interaction effect between B and C on the dust suppressant’s evaporation resistance. However, the 3D response surface plots for AB and AC show irregular slopes, suggesting that the interaction effects between A and B (Figure 6a) and A and C (Figure 6b) on the evaporation resistance are not significant despite a substantial color change from blue to red.

The contour lines for BC form an elliptical shape, signifying a significant interaction between AEO-13 and C_3_H_8_O_3_, whereas the contour lines for AB and AC appear irregular, indicating that the interaction effects between these factors are not significant. In Figure 6c, when the mass concentration of C_3_H_8_O_3_ is 2.2%, the evaporation resistance initially increases, then decreases, and the amplitude of the increase is substantial as AEO-13 concentration increases. The less pronounced interaction effects between AB and AC may be attributed to an increase in cross-linking density with rising solution concentration, forming a viscous gel-like structure. This alters the water retention mechanism of the dust suppressant, with HEC acting as a binding agent to form larger aggregates that trap water inside, while C_3_H_8_O_3_ forms a covering film on the particle surface. This interplay could influence their synergistic effects.

**Figure 7:**
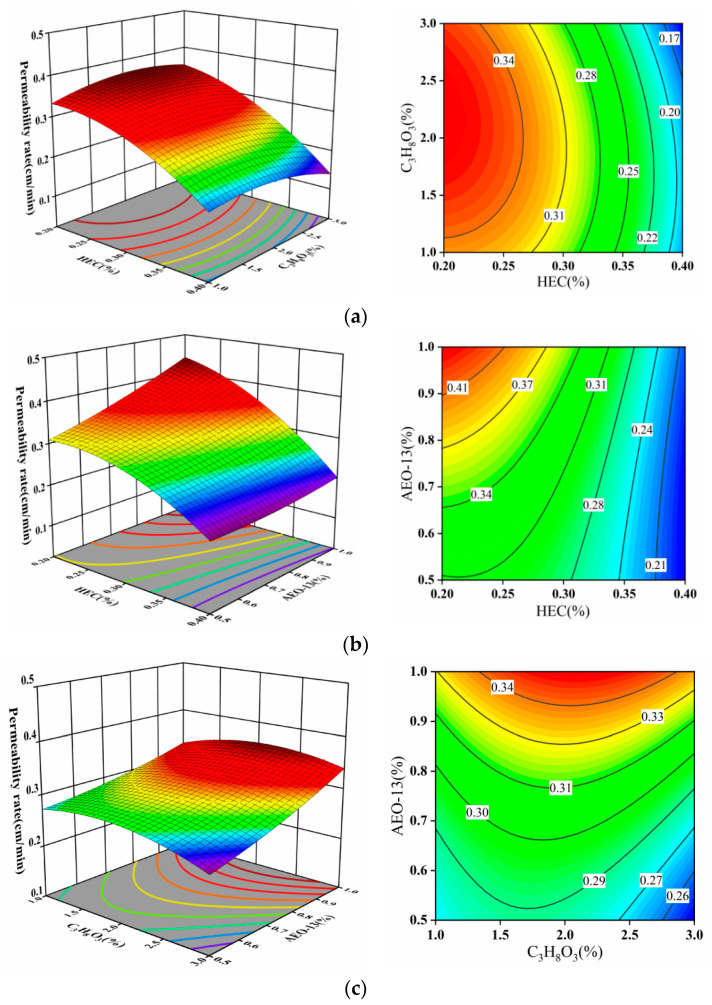
(**a**) HEC and C_3_H_8_O_3_; (**b**) HEC and AEO-13; (**c**) C_3_H_8_O_3_ and AEO-13 effects on permeability rate using 3D surface and contour plots.

The 3D surface plots in Figure 7 depict significant curvature in the response surface for permeability rate (AB and AC), with a substantial color change from blue to red. In contrast, the permeability rate response surface for BC exhibits a gentler slope, indicating a considerable impact of the interaction between hydroxyethyl cellulose (HEC) and propylene glycol (C_3_H_8_O_3_), AEO-13 on the permeability rate. The contour plots for AB form elliptical shapes, while those for AC and BC appear as incomplete ellipses, indicating a noticeable interaction between these factors. In Figure 7a, when the mass concentration of C_3_H_8_O_3_ is 2%, the permeability rate of the dust suppressant increases with the rise in HEC mass concentration, with the amplitude increasing after an initial modest change.

### 4.3. Optimization and Validation

#### 4.3.1. Model Validation

**Figure 8:**
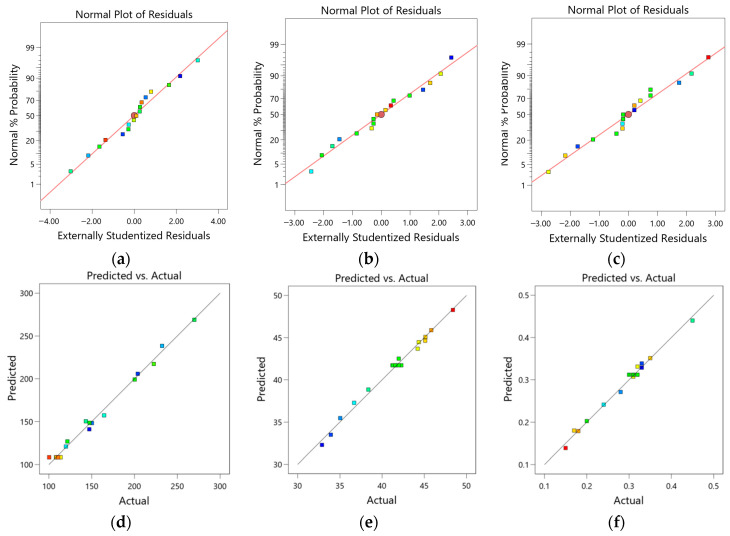
Normal probability distribution of residuals for (**a**) viscosity, (**b**) evaporation resistance, (**c**) permeability rate; and residuals against predicted values for (**d**) viscosity, (**e**) evaporation resistance, (**f**) permeability rate.

When assessing whether the model adheres to assumptions or detecting outliers, the concept of residuals, the difference between actual and fitted values, is commonly employed. The normal probability plots of residuals and the distribution plots of predicted versus actual values are presented in Figure 8. It is evident that the majority of experimental points are evenly distributed along or near the straight line, indicating that the residuals for the viscosity, evaporation resistance, and permeability rate models are random, independent of predictor variables, and follow a normal distribution. Moreover, there are few outliers, signifying good model adaptability. In Figure 8a–c, most points are uniformly distributed around the line P, indicating a close match between the predicted and observed values for all three models, affirming the rationality of the optimal ratios predicted by these models.

#### 4.3.2. Optimal Selection of Values

According to the requirements for the use of the composite dust suppressant, a viscosity value in the range of 100–120 mPa·s ensures effective bonding without causing nozzle blockage. For evaporation resistance, the goal is to maximize it, indicating better water retention and enhanced dust suppression. Regarding permeability rate, the optimization target is set within the range of 0.3–0.4 cm/min. This balances the need to prevent excessive permeability that might hinder bonding effectiveness while maintaining sufficient wetting properties for a broader dust suppression range.

Considering the interactive effects of various additives through software analysis, the optimal mass fractions for the composite dust suppressant solution are determined as follows: 0.2% for HEC, 2.097% for C_3_H_8_O_3_, and 0.693% for AEO-13. The predicted values from the response surface model for this composition are a viscosity of 106.7 mPa·s, evaporation resistance of 46.85%, and permeability rate of 0.35 cm/min. The measured values closely match with viscosity at 108.9 mPa·s, evaporation resistance at 45.78%, and permeability rate at 0.34 cm/min. These results, obtained at the optimal composition, provide valuable insights for practical application in engineering. The pH of the composite dust suppressant is 6.5, and the surface tension is 27.7 mN/m.

## 5. Characterization of Dust Suppressant Properties

### 5.1. SEM Analysis

**Figure 9:**
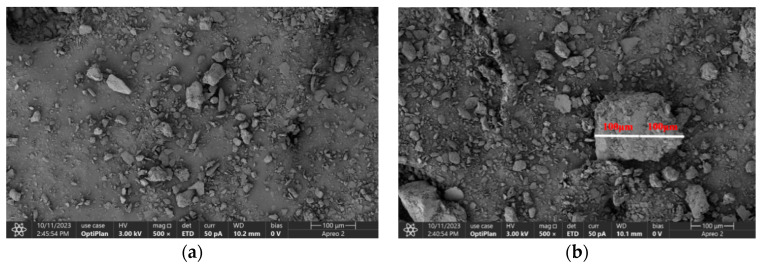
(**a**) Pure water; (**b**) dust suppressant treatment of soil samples.

In order to further observe the microstructure of the soil samples after spraying with the dust suppressant and achieve a comprehensive evaluation of the dust suppression effect, scanning electron microscopy (SEM) was conducted on soil samples sprayed with pure water and an equal amount of composite chemical dust suppressant [11]. The reason for using images scanned by a 500× electron microscope in Figure 9 is to demonstrate the particles bonded together after spraying the dust suppressant. If the magnification is too high, the phenomenon of particle agglomeration is not as apparent. This microscopic analysis allows for the observation of surface morphology changes in the soil samples. The experiment utilized a Thermo Fisher Apreo2C (Waltham, MA, USA) scanning electron microscope. The specific experimental steps were as follows: Two soil samples were prepared in evaporation dishes, and equal amounts of pure water and composite dust suppressant were evenly sprayed on the surfaces. After natural penetration, the samples were placed in a forced-air drying oven for drying. The electron microscope was set to a magnification of 500× for scanning, and the results are shown in Figure 9.

By comparing Figure 9a,b, it is evident that the surface particles of the soil sample sprayed with pure water are relatively scattered and not aggregated. In contrast, the soil sample sprayed with the composite chemical dust suppressant exhibits the formation of several larger substances, with the largest substance measuring approximately 200 μm in length. These aggregates are formed by binding numerous small particles together, indicating a significant binding effect of the dust suppressant. The surfaces of these aggregates are smooth, resembling a dense network that encapsulates the entire soil sample. This effectively traps moisture inside, enhancing the particles’ resistance to evaporation and contributing to dust suppression. In comparison, the soil sample treated with water does not show the formation of aggregates from multiple particles, indicating that after water spraying, moisture is prone to rapid evaporation, resulting in poor dust suppression. The observed microscopic changes affirm that the dust suppressant exhibits good dust suppression effects, particularly in terms of binding and evaporation resistance.

### 5.2. Toxicological Analysis

The toxicity testing of the composite chemical dust suppressant was outsourced to a specialized testing institution. In terms of odor, this dust suppressant exhibits no noticeable irritant smell and has a milky-white color, with no visible foreign mechanical impurities, complying with the Chinese standard TB/T3210.1-2009 [28]. Regarding the detection of toxic and hazardous substances such as heavy metals, the formaldehyde content in this dust suppressant is 0.9 mg/L, meeting the Chinese standard HJ601-2011 [29]. The total chromium content is <0.004 mg/L, the total cadmium content is <0.01 mg/L, and the total lead content is <0.05 mg/L, all in accordance with the Chinese standard GB/T7475-1987 [30]. The total arsenic and total mercury contents are 0.0006 mg/L and 0.00097 mg/L, respectively, fulfilling the requirements of the Chinese standard HJ694-2014 [31]. Note that as the soil samples used in this study are sourced from China and, considering that the experiments have only been conducted in construction areas in China due to current conditions, the toxicological standards referenced are based on domestic Chinese standards. However, in subsequent stages, international toxicological standards will be consulted for evaluation to enhance their applicability globally.

### 5.3. Wind Erosion Resistance Test

**Figure 10:**
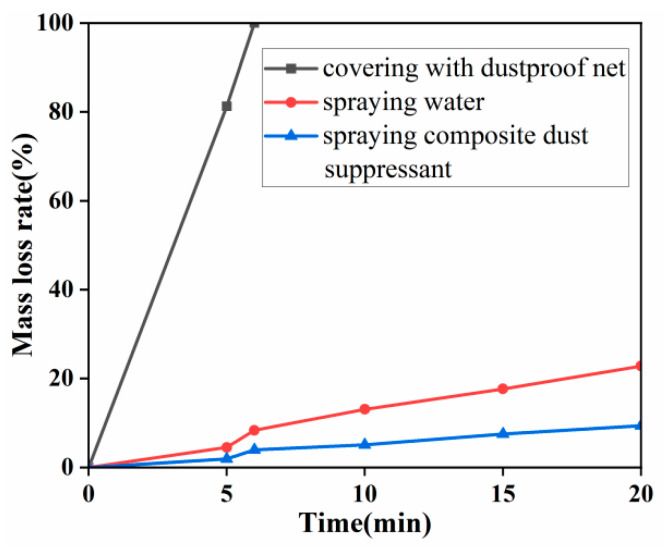
Mass loss rate tested by wind erosion.

The ability to resist natural wind erosion is a crucial indicator for assessing the practical usability of the composite dust suppressant. In this experiment, 500 g of dried soil was naturally spread into a 40 cm × 40 cm square on a glass plate. With a spraying rate of 2 L/m^2^, 0.32 L of the composite dust suppressant was evenly sprayed. After the soil dried slightly, its weight was measured and recorded as W. Two parallel control groups were set up, spraying water and covering with a dustproof net, respectively. The experiment used a blower to simulate natural wind at eleven-level wind force, continuously eroding for 20 min. The mass loss rate was used as an indicator to evaluate the effectiveness of wind erosion resistance. The experimental results are shown in Figure 10.

According to the experimental results in Figure 10, the resistance to natural wind erosion for the three dust suppression methods, from low to high, is as follows: covering with dustproof net < spraying water < spraying composite dust suppressant. Under the erosion of the blower, the mass loss rate of the sample covered with dustproof net reached 100% around 6 min, while the samples sprayed with water and composite dust suppressant, under the conditions of eleven-level natural wind, had mass loss rates of 22.82% and 9.36%, respectively, after 20 min of erosion. These rates are much lower than the mass loss rate of the dustproof net-covered sample, indicating that the composite chemical dust suppressant has excellent resistance to wind erosion.

### 5.4. Applications: On-Site Spraying Test

**Figure 11:**
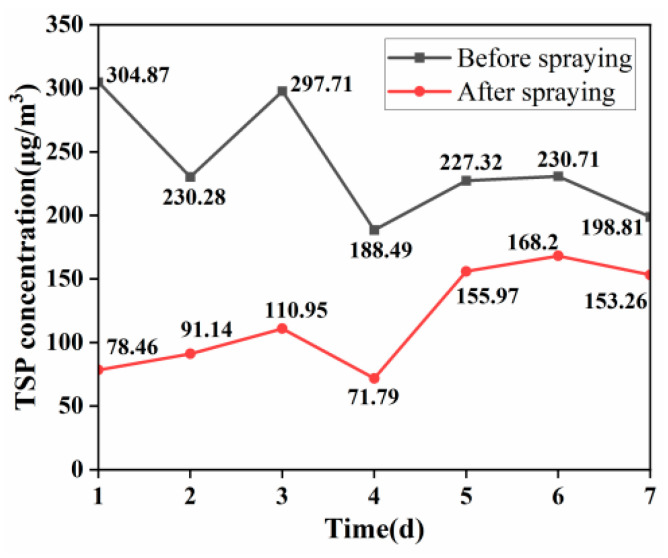
Variation in TSP concentration.

To visually assess the actual effectiveness of the dust suppressant, further spraying experiments were conducted at a construction site in Chengdu, China. An environmental air sampler was used to collect total suspended particles (TSPs) in the air at the construction site. The sampling point was located in an area adjacent to the main road for vehicle transport at the construction site. This area served as the only passage for vehicles entering and exiting the construction site daily and was situated close to the site’s temporary activity buildings. Besides dust generated during construction activities, it could also capture dust generated by human activities. TSP concentration data were collected for 14 days, with the first 7 days representing TSP concentrations at the construction site before the application of the dust suppressant and the subsequent 7 days reflecting the changes in TSP concentrations after the application of the dust suppressant, as illustrated in Figure 11.

The comparison of TSP concentrations before and after sampling reveals significant findings. Prior to the application of the dust suppressant, the average 24 h emission concentration of total suspended particles (TSPs) at the construction site was 239.74 μg/m^3^. During the sampling period, the highest 24 h emission concentration of TSP occurred on the first day, reaching 304.87 μg/m^3^. Additionally, for 5 days, the 24 h emission concentration of TSP exceeded 200 μg/m^3^. Over the continuous 7-day sampling period, the 24 h emission concentration of TSP at this construction site exceeded the Chinese second-level standard on one day and did not comply with the Chinese first-level standard for 5 days [32]. The TSP concentration remained relatively high.

After the on-site trial of the composite dust suppressant, the average 24 h emission concentration of TSP at the construction site was reduced to 118.54 μg/m^3^. The highest recorded value during the sampling period occurred on the sixth day, with a concentration of 168.20 μg/m^3^. Importantly, all values met the Chinese standard, indicating a notable decrease in TSP concentration compared to the pre-application period. This reflects the suppressive effect of the composite dust suppressant on airborne particulate matter. However, due to limited resources, the dust suppressant is currently only being tested at construction sites in Chengdu, China. Future research will aim to conduct experimental studies in different regions.

## 6. Conclusions

Through single-factor experiments, response surface optimization experiments, response value variance analysis, model validation, scanning electron microscopy, and toxicity analysis, this study explored the optimal combination of various additives in the composite chemical dust suppressant. A comparison analysis of the dust suppression performance of each additive yielded the following main conclusions:

1. Optimal components include hydroxyethyl cellulose, glycerol, and Isotridecanol polyoxyethylene ether. HEC ensures stable viscosity, glycerol enhances water retention, and AEO-13 acts as a surfactant. HEC and sodium polyacrylate have viscosity values of 137.48 mPa·s and 297.7 mPa·s, respectively, at a mass concentration of 0.5%. Glycerol and triethanolamine at a mass concentration of 3% have water retention rates of 12.83% and 9.1%, respectively. Isostearyl alcohol polyoxyethylene ether exhibits the lowest surface tension at 29.5 mN/m and thus is selected as the surfactant.
2. Applying response surface methodology, the ideal composition is 0.2% HEC, 2.097% glycerol, and 0.693% AEO-13. This model was verified, confirming its effective predictive accuracy.
3. The resulting dust suppressant exhibits a viscosity of 108.9 mPa·s, an anti-evaporation rate of 45.78%, a permeability rate of 0.34 cm/min, a pH of 6.5, and a surface tension of 27.7 mN/m.
4. Scanning electron microscopy (SEM) analysis reveals improved surface morphology, forming denser aggregates and validating the dust suppressant’s ability to protect moisture and bond fine particles.
5. After conducting on-site spraying experiments at the construction site, data collected from TSP indicate that the weekly average concentration of TSP before spraying at the construction site was 239.74 μg/m^3^. Following spraying, the weekly average concentration of TSP decreased to 118.54 μg/m^3^, representing a 51% reduction compared to before spraying. This demonstrates the dust suppression effect of the dust suppressant on particulate matter. However, due to limited resources, the dust suppressant is currently only being tested at construction sites in Chengdu, China. Future research will aim to conduct experimental studies in different regions.
6. This dust suppressant has room for further optimization in terms of raw material selection, aiming to reduce its preparation cost. Moreover, under conditions of temperatures exceeding 50 °C and gale-force wind speeds surpassing level 9, the dust suppression efficiency of the agent can significantly decrease. To ensure its dust-suppression effectiveness, multiple spraying applications can be employed.

## Figures and Tables

**Table 1:** Measurement of water retention rate using glycerol solution.

Time (h)	Water Content (%)	0.01%	0.05%	0.1%	0.5%	1%	3%
0	100	100	100	100	100	100	100
24	86.41	87.41	87.76	88.39	88.31	88.04	87.62
48	71.74	73.16	73.74	75.21	75.21	74.47	72.99
72	55.92	58.91	59.99	61.32	61.32	60.5	58.09
96	32.59	38.24	40.08	40.83	40.83	40.21	38.56
120	10.01	16.43	19.71	20.09	19.7	19.91	20.44
144	2.37	5.63	9.13	9.78	9.82	10.6	12.83

**Table 2:** Measurement of water retention rate using triethanolamine solution.

Time (h)	Water Content (%)	0.01%	0.05%	0.1%	0.5%	1%	3%
0	100	100	100	100	100	100	100
24	86.41	87.62	87.45	87.78	87.04	87.6	86.38
48	71.74	74.03	73.16	73.6	72.14	72.76	70.67
72	55.92	59.59	58.8	58.81	57.29	57.84	54.69
96	32.59	38.42	38.23	37.54	35.87	36.48	33.99
120	10.01	16.44	17.01	15.46	14	15.82	16.25
144	2.37	5.79	6.92	5.47	4.75	6.87	9.1

**Table 3:** Details of mix design using RSM.

Std	Run	HEC (%)	C_3_H_8_O_3_ (%)	AEO-13 (%)	Viscosity (mPa·s)	Evaporation Resistance (%)	Permeability Rate (cm/min)
8	1	0.4	2	1	269.9	41.97	0.2
5	2	0.2	2	0.5	119.9	44.22	0.31
11	3	0.3	1	1	200.2	32.88	0.33
14	4	0.3	2	0.75	113.6	41.56	0.3
7	5	0.2	2	1	143.1	38.37	0.45
2	6	0.4	1	0.75	203.8	45.83	0.18
4	7	0.4	3	0.75	232.2	48.39	0.15
1	8	0.2	1	0.75	147.2	44.35	0.32
13	9	0.3	2	0.75	108.3	41.23	0.32
10	10	0.3	3	0.5	147.5	36.69	0.24
16	11	0.3	2	0.75	110.8	42.28	0.32
9	12	0.3	1	0.5	121.6	35.04	0.28
17	13	0.3	2	0.75	100.3	41.98	0.31
12	14	0.3	3	1	222.5	33.92	0.33
6	15	0.4	2	0.5	164.5	45.11	0.17
15	16	0.3	2	0.75	109.1	41.56	0.31
3	17	0.2	3	0.75	150.3	45.13	0.35

**Table 4:** Variance analysis of viscosity.

Source	Sum of Squares	Degree of Freedom	Mean Square	F-Value	*p*-Value	Significance
Model	41,409.15	9	4601.02	92.76	<0.0001	**
A	12,004.75	1	12,004.75	242.03	<0.0001	**
B	794.01	1	794.01	16.01	0.0052	**
C	9954.6	1	9954.6	200.7	<0.0001	**
AB	160.02	1	160.02	3.23	0.1155	
AC	1689.21	1	1689.21	34.06	0.0006	**
BC	3.24	1	3.24	0.0653	0.8056	
A^2^	6136.93	1	6136.93	123.73	<0.0001	**
B^2^	5695.09	1	5695.09	114.82	<0.0001	**
C^2^	3242.95	1	3242.95	65.38	<0.0001	**
Residuals	347.2	7	49.6			
Lack of fit	248.29	3	82.76	3.35	0.1368	
Pure error	98.91	4	24.73			
Total deviation	41,756.34	16				

Note: R^2^ = 0.9917, AP = 29.7126, ** indicates high significance.

**Table 5:** Variance analysis of evaporation resistance.

Source	Sum of Squares	Degree of Freedom	Mean Square	F-Value	*p*-Value	Significance
Model	311.26	9	34.58	86.66	<0.0001	**
A	10.65	1	10.65	26.68	0.0013	*
B	4.55	1	4.55	11.39	0.0118	*
C	24.22	1	24.22	60.69	0.0001	**
AB	0.7921	1	0.7921	1.98	0.2017	
AC	1.84	1	1.84	4.6	0.0691	
BC	0.093	1	0.093	0.2331	0.644	
A^2^	151.28	1	151.28	379.06	<0.0001	**
B^2^	13.51	1	13.51	33.84	0.0007	**
C^2^	118.21	1	118.21	296.2	<0.0001	**
Residuals	2.79	7	0.3991			
Lack of fit	2.12	3	0.707	4.21	0.0995	
Pure error	0.6725	4	0.1681			
Total deviation	314.05	16				

Note: R^2^ = 0.9911, AP = 32.9945, * indicates significance, ** indicates high significance.

**Table 6:** Variance analysis of permeability rate.

Source	Sum of Squares	Degree of Freedom	Mean Square	F-Value	*p*-Value	Significance
Model	0.0921	9	0.0102	79.14	<0.0001	**
A	0.0666	1	0.0666	515.23	<0.0001	**
B	0.0002	1	0.0002	1.55	0.2536	
C	0.012	1	0.012	92.91	<0.0001	**
AB	0.0009	1	0.0009	6.96	0.0335	*
AC	0.003	1	0.003	23.4	0.0019	**
BC	0.0004	1	0.0004	3.09	0.122	
A^2^	0.0058	1	0.0058	45.19	0.0003	**
B^2^	0.0026	1	0.0026	19.95	0.0029	**
C^2^	0.0003	1	0.0003	1.96	0.2046	
Residuals	0.0009	7	0.0001			
Lack of fit	0.0006	3	0.0002	2.98	0.1597	
Pure error	0.0003	4	0.0001			
Total deviation	0.093	16				

Note: R^2^ = 0.9903, AP = 34.5443, * denotes significance, ** denotes high significance.

## Data Availability

Data will be made available on request.
